# The role of empathy in the coach-athlete relationship in cheerleading

**DOI:** 10.1192/j.eurpsy.2023.2160

**Published:** 2023-07-19

**Authors:** O. Krykun, V. Voronova, S. Fedorchuk

**Affiliations:** National University of Ukraine on Physical Education and Sport, Kyiv, Ukraine

## Abstract

**Introduction:**

In 2016, the International Olympic Committee (IOC) preliminarily recognized cheerleading as an Olympic sport. In this regard, the participation of Ukrainian cheerleaders in the 2024 Olympics in Paris is quite likely. The athlete’s performance depends on both physical and psychological factors. Human behavior depends on interaction with other people. Empathy is based on social interactions and is defined as understanding, awareness, sensitivity, and the ability to experience the feelings, thoughts, and experiences of another person. There is little research on the impact of empathy on individual athlete and team performance and performance. Psychological training in sports involves a significant amount of work with the athlete, in particular on the part of the coach: the formation of personality and interpersonal relations, the development of sports intelligence, mental functions and psychomotor qualities, etc.

**Objectives:**

The purpose of the study was to determine the level of empathy among cheerleading coaches.

**Methods:**

The following research methods were used to realize the goal of the work: 1) theoretical analysis and generalization of literary sources and Internet data; 2) observation, questionnaire, interview; 3) method of expert evaluations; 4) method of diagnosing the level of empathy; 5) methods of non-parametric statistics.

**Results:**

According to the general (total) indicator of empathy, the vast majority of cheerleading coaches (n=18) fell into the group with a reduced level of empathy, which is generally characteristic of coaches of some other team sports. According to the results of the research, the following features were revealed regarding individual trends in the structure of empathy of the subjects. In particular, the lowest number of points for coaches was found on the scales “Rational component of empathy”, “Identification abilities (ability to imitate)” and “Intuition”.

**Conclusions:**

The data of the conducted research show that the vast majority of the surveyed coaches are characterized by a reduced level of empathy. The most pronounced components of empathy, according to the obtained results, were such components of the coaches’ personality as penetrating abilities (ease of establishing communicative ties), emotional sensitivity and instructions that promote or hinder empathy.

**Disclosure of Interest:**

None Declared

